# *In vitro* Antifungal Susceptibility Profiles of *Cryptococcus neoformans* var. *grubii* and *Cryptococcus gattii* Clinical Isolates in Guangxi, Southern China

**DOI:** 10.3389/fmicb.2021.708280

**Published:** 2021-08-10

**Authors:** Najwa Al-Odaini, Xiu-ying Li, Bing-kun Li, Xing-chun Chen, Chun-yang Huang, Chun-ying Lv, Kai-su Pan, Dong-yan Zheng, Yan-qing Zheng, Wan-qing Liao, Cun-wei Cao

**Affiliations:** ^1^Department of Dermatology and Venerology, First Affiliated Hospital, Guangxi Medical University, Nanning, China; ^2^Guangxi Health Commission Key Lab of Fungi and Mycosis Research and Prevention, Nanning, China; ^3^The People’s Hospital of Guangxi Zhuang Autonomous Region, Nanning, China; ^4^Fourth People’s Hospital of Nanning, Nanning, China; ^5^Shanghai Key Laboratory of Medical Fungal Molecular Biology, Second Military Medical University, Shanghai, China

**Keywords:** *Cryptococcus gattii*, variant identification, *in vitro* drug sensitivity, *C. neoformans* var. *grubii*, Guangxi

## Abstract

This study analyzed the *in vitro* drug sensitivity of *Cryptococcus* spp. from Guangxi, Southern China. One hundred three strains of *Cryptococcus* were recovered from 86 patients; 14 were HIV positive and 72 were HIV negative. Ninety-two strains were identified as *Cryptococcus neoformans* var. *grubii*, while 11 strains were identified as *Cryptococcus gattii* (5 *C. gattii sensu stricto* and 6 *Cryptococcus deuterogattii*). The recovered strains were tested against commonly used antifungal drugs (fluconazole, amphotericin B, 5-fluorocytosine, itraconazole, and voriconazole) and to novel antifungal drugs (posaconazole and isavuconazole) using CLSI M27-A4 method. The results showed that all isolates were susceptible to most antifungal drugs, of which the minimum inhibitory concentration (MIC) ranges were as follows: 0.05–4 μg/ml for fluconazole, 0.25–1 μg/ml for amphotericin B; 0.0625–2 μg/ml for 5-fluorocytosine, 0.0625–0.25 μg/ml for itraconazole, 0.0078–0.25 μg/ml for voriconazole, 0.0313–0.5 μg/ml for posaconazole, 0.0020–0.125 μg/ml for isavuconazole for *C. neoformans* var. *grubii* isolates, and 1–16 μg/ml for fluconazole, 0.125–1 μg/ml for 5-fluorocytosine, 0.25–1 μg/ml for amphotericin B, 0.0625–0.25 μg/ml for itraconazole, 0.0156–0.125 μg/ml for voriconazole, 0.0156–0.25 μg/ml for posaconazole, and 0.0078–0.125 μg/ml for isavuconazole for *C. gattii* isolates. Furthermore, some *C. neoformans* var. *grubii* isolates were found to be susceptible-dose dependent to 5-fluorocytosine and itraconazole. In addition, a reduction in the potency of fluconazole against *C. gattii* is possible. We observed no statistical differences in susceptibility of *C. neoformans* var. *grubii* and *C. gattii* in the tested strains. Continuous observation of antifungal susceptibility of *Cryptococcus* isolates is recommended to monitor the emergence of resistant strains.

## Introduction

Cryptococcosis is a common opportunistic invasive fungal infection caused mainly by *Cryptococcus neoformans* (*C. neoformans*, serotypes A, AD, and D) and *Cryptococcus gattii* (*C. gattii* serotypes B and C) (Guinea et al., 2010). The latter mainly affects otherwise healthy individuals, whereas *C. neoformans* is more common in immunocompromised patients (Park et al., 2009; Guinea et al., 2010; Maziarz and Perfect, 2016). Although the incidence of cryptococcosis has declined with the introduction of highly active antiretroviral therapy (HAART), immunocompromised individuals remain at risk, and mortality rate remain unacceptably high despite the continued research on cryptococcosis (Park et al., 2009; Selb et al., 2019). *C. neoformans* and *C. gattii* are genetically related to each other; however, they vary in the ecological niche geographic distribution, natural habitat, host infectivity, and pathogenicity (Gutch et al., 2015). For instance, *C. neoformans* has a global distribution, while *C. gattii* strains are more common in North America and Australia (Ellis and Pfeiffer, 1990). Moreover, *C. gattii* seems to be less sensitive to therapy and requires more aggressive management than *C. neoformans* (Speed and Dunt, 1995). China has the worlds’ largest population with large humid tropical and subtropic regions rich with vegetation, a climate favorable for the growth and spread of fungi. In addition, the number of immunocompromised (HIV and non-HIV) populations in China has been increasing in the past several decades, leading to an increase in the incidence and prevalence of aggressive fungal infections such as cryptococcosis, which is a heavy burden to public health (Fang et al., 2015). Furthermore, cryptococcosis can be easily misdiagnosed due to the vague and diversity of the clinical manifestations, leading to the delay of proper treatment. Therefore, early diagnosis and timely treatment play an important role in the prognosis of the disease. Several studies have been performed to investigate the microbiological, epidemiological, and clinical characteristics of *C. neoformans* and *C. gattii* strains in China (Chen et al., 2008, 2018; Feng et al., 2008; Wu et al., 2015, 2021; Fang et al., 2020; Jin et al., 2020; Xu et al., 2021). Unfortunately, relatively little is known about the pathogenic *Cryptococcus* species and their sensitivity to antifungal chemotherapy in Guangxi, Southern China. Guangxi province is located in the subtropical zone with a warm, humid climate where cryptococcosis is significantly common but might be severely underreported. Recently, new antifungal agents have been introduced, suggesting that the antifungal susceptibility profiles need to be researched and updated. Therefore, the current study aims to analyze the clinical characteristics of *Cryptococcus* infection and *in vitro* drug susceptibility of *Cryptococcus* spp. in Guangxi, Southern China.

## Materials and Methods

### Ethics Statement

This study was approved by the Medical Ethics Committee of the First Affiliated Hospital of Guangxi Medical University. The clinical data in this study were obtained with written consent from the patients or their families, and data collected concerning them was anonymized.

### Isolates and Clinical Data

Between May 2014 and May 2018, clinical isolates of *Cryptococcus* spp. from patients admitted to the First Affiliated Hospital of Guangxi Medical University, Nanning, Guangxi, the Fourth People’s Hospital of Nanning, and People’s Hospital of Guangxi Zhuang Autonomous Region were collected and recovered for this study. We assessed the patients’ medical records to collect clinical information. Strains with insufficient clinical data of patients were excluded.

### Strains Identification

#### Activation and Identification

A total of 120 *Cryptococcus* strains were taken out of the refrigerator at −80°C, resuscitated at 20°C for 24 h, transferred to Sabouraud dextrose agar (SDA) medium, and incubated at 27°C for 48–72 h, then transferred onto L-canavanine-glycine-bromothymol blue (CGB) medium for 3–7 days to differentiate *C. neoformans* from *C. gattii* species as previously mentioned (Klein et al., 2009). Three consecutive purifications were made using the streak plate technique to ensure the growth and purity of the strains. Multiple colonies were transferred to SDA medium for cultivation at 27°C and enrichment for later use.

#### Extraction of *Cryptococcus* Neoformans Protein

A sterile loop full of the purified isolated strains (about 5 mg) was added to an Eppendorf tube containing 300 μl of distilled H_2_O and was mixed thoroughly. Nine hundred microliters of absolute ethanol was added to the tube, mixed, and centrifuged at 12,000 r/min for 2 min. After discarding the supernatant, the tube was centrifuged again at 12,000 r/min for 2 min and later placed at room temperature to dry. Following the addition of 50 μl of 70% formic acid and 50 μl of acetonitrile, the tube was centrifuged for 2 min at 12,000 r/min. The supernatant was then transferred to a new tube for use.

#### Matrix-Assisted Laser Desorption Ionization-Time of Flight Mass Spectrometry Identification

The samples were overlaid with 1 μl of matrix solution consisting of a saturated solution of α-cyano-4-hydroxycinnamic acid in 50% acetonitrile–2.5% trifluoroacetic acid and again allowed to air dry prior to analysis. For each isolate, a spectrum with a mass-to-charge range of 2,200–22,000 Da was generated as an average of 240 laser shots in an automatic acquisition mode. When poor spectra (fewer than 10 well-defined peaks above 1,000 arbitrary units) were obtained, analysis was repeated with an extra wash step during the protein extraction procedure, which improved the quality of the spectra. The MALDI Biotyper 3.0 database was used to compare the data and record the mass spectrometry identification results. According to the instruction manual, a log score ≥1.70 indicates correct identification; a log score <1.70 indicates that the identification is incorrect or unable to be identified (McTaggart et al., 2011).

#### DNA Extraction and ITS Sequencing

As a reference “gold standard,” 20 strains of *Cryptococcus* were randomly selected to extract DNA for ITS sequencing. Strains were cultured on SDA medium at 27°C for 72 h for DNA extraction according to the operation steps of the fungal genomic DNA extraction kit produced by Jiangsu Kangwei Century Biotechnology Co., Ltd (Changping, Beijing, China). According to the operating manual, the concentration of genomic DNA is determined using a nucleic acid protein analyzer and then placed in a refrigerator at −20°C for later use. Ribosomal RNA (rRNA) internal transcribed spacer Region (rITS) sequencing is performed on the extracted DNA using primer sets (upstream primer sequence ITS1: 5′-TCCGTAGGTGAACCTGCGG-3′) and (downstream primer sequence ITS4: 5′-TCCTCCGCTTATTGATATGC-3′). A total volume of 50 μl (Taq-Mix, 25 μl; upper material, 2 μl; downstream primer, 2 μl; at least 100 ng of DNA extract and sterile deionized water) was used to perform polymerase chain reaction (PCR) for 35 cycles at 95°C with 3 min initial denaturation, 30 s denaturation at 95°C, 30 s annealing at 55°C, 2 min extension at 72°C, and final extension cycle for 10 min at 72°C, and then stored at 4°C. A total of 6 μl of PCR products was separated slowly into 1% agar gel spotting hole electrophoresis at 120 V for 30 min. The agar gel was placed under the UV gel imager to observe whether the PCR product and marker are clear and record the band length. PCR products are then sent with clear bands under electrophoresis to Guangzhou Kinco Biotechnology Co., Ltd. for two-way sequencing. Results were uploaded to the National Center for Biotechnology Information (NCBI) Genebank database^1^ for comparison and identification.

### Antifungal Susceptibility Test

#### Antifungal and Suspensions Preparation

The antifungal susceptibility testing was assessed by the checkerboard broth microdilution method performed according to the Clinical and Laboratory Standards Institute (CLSI) protocol M27-A4 (Clinical and Laboratory Standards Institute (CLSI), 2017). The minimum inhibitory concentration (MIC) value was determined for fluconazole (FLC), amphotericin B (AmB), 5-fluorocytosine (5-FC), itraconazole (ITC), voriconazole (VOC), posaconazole (POS), and isavuconazole (ISA). Antifungal drugs were provided as powders with known potency from Sigma Chemical Co. (St. Louis, MO, United States). Stock solutions were prepared as follows: FLC and 5-FC were dissolved in sterile distilled water to a drug storage solution of 1,280 μg/ml, while AmB, ITC, VOC, POS, and ISA were dissolved in dimethyl sulfoxide (DMSO) (Sigma Chemical Co., United States) into a stock solution of 1,600 μg/ml and stored at −20°C until needed. The final concentration ranges were 0.125–64 μg/ml for FLC, 0.00156–8 μg/ml for AmB and 5-FC, 0.0020–1 g/ml for ITC, VOC, POS, and ISA, respectively. The yeast inocula were adjusted to a concentration of 1 × 10^3^–5 × 10^3^ cfu/ml in Roswell Park Memorial Institute (RPMI) 1640 medium as measured by a hemocytometer, and an aliquot of 0.1 ml was added to each well containing various concentrations of antifungal drugs. The 96-well plates were incubated at 37°C. The assays were read 72 h after inoculation. *Candida parapsilosis* ATCC 22019 was used as a quality control strain for susceptibility tests.

#### MIC and MIC Interpretations

According to the CLSI M27-A4 protocol, the MIC of AmB was defined as the lowest concentration that produced complete growth inhibition, while the MIC for other antifungal agents were defined as the lowest concentrations at which there was 50% inhibition of growth (≥50%) compared with that of drug-free control (optical clear). The MIC_50_ and MIC_90_, on the other hand, are the concentrations capable of inhibiting the growth of isolates by 50 and 90%, respectively (Favalessa et al., 2014). The interpretive MIC criteria for FLC were as follows: susceptible (S), ≤8 μg/ml; susceptible-dose dependent (SDD), 16–32 μg/ml; and resistance (R), ≥64 μg/ml; for 5-FC, ≤4 μg/ml (S), 8–16 μg/ml (SDD), and ≥32 μg/ml; for ITC, ≤0.125 μg/ml (S), 0.25–0.5 μg/ml (SDD), and ≥1 μg/ml; for VOR, ≤1 μg/ml (S) based on previous studies (Pfaller et al., 2005; Dias et al., 2006; Bertout et al., 2013; Gutch et al., 2015). For *Cryptococcus*, interpretative criteria have not been defined for POS, ISA, and AmB; hence, data available for *Candida* spp. were used, as previously reported (Revankar et al., 1988; Rodríguez-Tudela et al., 1995; Pfaller et al., 1999). Based on the recommendation of previous studies, the ECVs for *C. neoformans* var. *grubii* were 8.0 μg/ml for FLC, 1.0 μg/ml for AmB, 4 μg/ml for 5FC, 0.125 μg/ml for ITC, 1.0 μg/ml for VOR, 0.5 μg/ml for POS, and 0.25 μg/ml for ISA; and that for *C. gattii* were 8 μg/ml for FLC, 4 μg/ml for 5FC, 0.5 μg/ml for AmB, ITC, VOR, and POS, and 0.25 μg/ml for ISA (Espinel-Ingroff, 2012; Espinel-Ingroff et al., 2012, 2015; Lockhart et al., 2012). The susceptibility of each *Cryptococcus* spp. isolate was determined in triplicate at different times for optimal results.

### Statistical Analysis

Statistical analysis was performed using SPSS 17.0 (SPSS Inc., Chicago, IL, United States) where *p* < 0.05 was considered statistically significant.

## Results

### Isolates and Clinical Data

**FIGURE 1:**
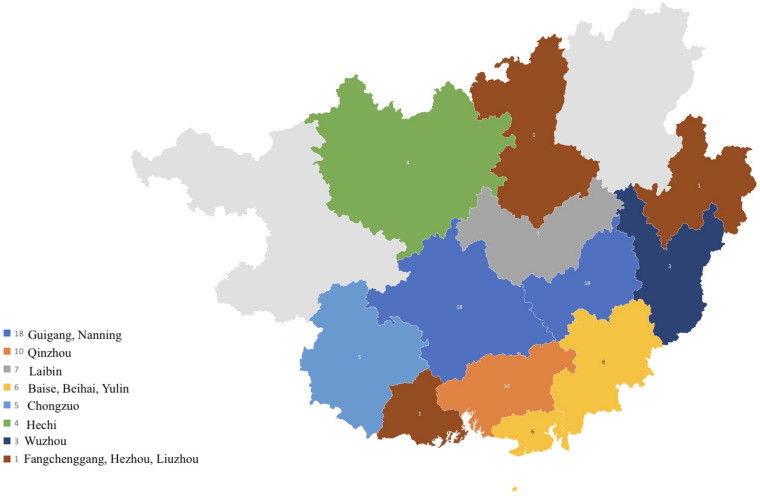
Geographic distribution of cryptococcosis patients from Guangxi province, Southern China.

A total of 103 strains from 86 patients were included in this study (Table 1). Fifty-nine of them were male, and twenty-seven were female. The age distribution ranged from 21 to 84 years. Of the 86 patients, 14 were HIV positive and 72 were HIV negative. A total of 55 had no underlying diseases, 21 had liver diseases, 7 were diabetic, 4 had systemic lupus erythematosus (SLE), 1 had a history of immunosuppressing therapy, 3 had liver cancer, and 1 had rheumatoid arthritis. All 86 patients came from 13 different cities in Guangxi province, Southern China (Figure 1). Of the 103 strains, 17 were episode strains (i.e., strains recovered from different parts of the same patient simultaneously or from the same part at different times). In total, 91 were isolated from cerebrospinal fluid, 7 were from lung tissue, 3 were from alveolar lavage, and 2 from the skin.

**TABLE 1:** Demographic and clinical characteristics of the 86 patients with cryptococcosis.

Variables	*C. neoformans* var. *grubii* (*n* = 75)	*Cryptococcus gattii* (*n* = 11)
		
Age, year, average	46.58	38.09
Gender, male	51	8
HIV (+), *n*	14	–
**Underlying diseases**		
None, *n*	51	4
Diabetes mellitus, *n*	7	–
HBV, *n*	13	5
HCV, *n*	1	2
Liver diseases^*a*^, *n*	4	1
Liver cancer, *n*	3	–
Corticosteroids administration, *n*	1	–
Autoimmune diseases^*b*^, *n*	4	1
**Clinical diagnosis**		
Cryptococcal meningitis	47	10
Pulmonary cryptococcosis	8	1
Disseminated cryptococcosis	20	–
**Clinical symptoms**		
Neurological abnormalities^*c*^, *n*	31	1
Headache, *n*	61	9
Fever, *n*	51	9
Cough, *n*	13	2
Vomiting, *n*	39	6
Abnormal vision, *n*	2	2
Skin lesions, *n*	1	–
**Treatment outcome**		
Survived, *n*	47	10
Died, *n*	27	1
Unknown, *n*	1	–
**Specimen source**	**(*n* = 92)**	**(*n* = 11)**
CSF, *n*	81	10
Lung tissue, *n*	6	1
BALF, *n*	3	–
Skin tissue, *n*	2	–

*n, number; HBV, hepatitis B virus; HCV, hepatitis C virus; CSF, cerebrospinal fluid; BALF, bronchoalveolar lavage fluid.*

*^*a*^Liver cirrhosis, fatty liver.*

*^*b*^Systemic lupus erythematous, rheumatoid arthritis.*

*^c^Convulsion, altered consciousness, epilepsy.*

### Strain Identification

Eleven strains successfully turned canavanine-glycine-bromothymol blue (CGB) medium to blue and were consequently identified as *C. gattii*. The remaining 92 strains were identified as *C. neoformans* var. *grubii* according to the MALDI-TOF MS technique, with mass spectrum scores ≥1.7. The DNA results on the database comparison result were 100% consistent with the MALDI-TOF MS identification result, making the results of MALDI-TOF MS in our study credible. The identification of *C. gattii* strains and their genotypes were analyzed in detail in our previous study using multilocus sequence typing (MLST) technique, where five isolates were identified as *C. gattii sensu stricto* (*C. gattii s.s*.), and six were identified as *C. deuterogattii* serotype (Huang et al., 2020).

### Drug Susceptibility Results

The seven antifungal agents tested retained activity against all isolates. The MIC, MIC_50_, and MIC_90_ values are presented in Table 2.

**TABLE 2:** Antifungal susceptibilities for 130 *Cryptococcus* isolates.

Isolates and antifungal	MIC range	GM	MIC_50_	MIC_90_
	
	(μg/ml)	(μg/ml)	(μg/ml)	(μg/ml)
***C. neoformans* var. *grubii* (*n* = 92)**				
FLC	0.05–4	1.57	2	4
AmB	0.25–1	0.75	1	1
5-FC	0.0625–2	0.57	0.5	1
ITC	0.0625–0.25	0.08	0.0625	0.125
VOC	0.0078–0.25	0.03	0.0313	0.0625
POS	0.0313–0.5	0.13	0.125	0.25
ISA	0.0020–0.125	0.02	0.0156	0.0625
***Cryptococcus gattii* (*n* = 11)**				
FLC	1–16	2.57	2	4
AmB	0.25–1	0.47	0.5	0.5
5-FC	0.125–1	0.28	0.25	0.5
ITC	0.0625–0.25	0.18	0.25	0.25
VOC	0.0156–0.125	0.05	0.0625	0.125
POS	0.0156–0.25	0.07	0.0625	0.25
ISA	0.0078–0.125	0.04	0.0625	0.125

*MIC_50_ and MIC_90_, the concentration capable of inhibiting the growth of isolates by 50 and 90%, respectively. GM, geometric means.*

#### C. neoformans var. grubii

The MIC ranges of the 92 *C. neoformans* var. *grubii* strains for the 7 drugs were as follows: FLC, 0.05–4 μg/ml; AmB, 0.25–1 μg/ml; 5-FC, 0.0625–2 μg/ml; ITC, 0.0625–0.25 μg/ml; VOC, 0.0078–0.25 μg/ml; POS, 0.0313–0.5 μg/ml; and ISA, 0.0020–0.125 μg/ml. All 92 clinical strains of *C. neoformans* var. *grubii* were susceptible to FLC, AmB, VOC, POS, and ISA; 91 strains (98.91%) were susceptible to 5-FC, while 1 strain (1.09%) was susceptible-dose dependent; 85.87% (79/92) of the strains were susceptible to ITC; and the remaining 14.13% (13/92) strains were susceptible-dose dependent to ITC.

#### C. gattii

Similar to *C. neoformans* var. *grubii*, none of the strains demonstrated resistance to the antifungal drugs (Table 2). However, we observed an MIC value above the ECVs to FLC against a single *C. deuterogattii* isolate.

## Discussion

This study analyzed the epidemiology and *in vitro* antifungal susceptibility profiles of *Cryptococcus* spp. in a large-scale population from Guangxi, Southern China. Consistent with previous reports from China, our study showed that the prevalence of cryptococcosis caused by *C. neoformans* is higher than those caused by *C. gattii* (Chen et al., 2008; Fang et al., 2015; Wu et al., 2015). In addition, the disease was more observed among otherwise healthy individuals. Furthermore, our study demonstrated that *C. neoformans* was responsible for the infection in immunocompetent, which is contrary to the HIV-associated cryptococcosis cases reported in the United States, Africa, and Europe (Dromer et al., 1996; Moosa and Coovadia, 1997; Hajjeh et al., 1999). Similar to reports from other parts of China, *C. neoformans* var. *grubii* was the dominant pathogenic strain in Guangxi (Chen et al., 2008).

In recent years, MALDI-TOF MS has emerged as a rapid, accurate, and cost-effective method for microbial identification and diagnosis (Singhal et al., 2015). Additionally, MALDI-TOF MS can replace some traditional identification methods, which improves clinical diagnosis and treatment. Furthermore, MALDI-TOF MS can help predict drug-resistant fungal isolates by identifying inflicting fungal species (McTaggart et al., 2011). Moreover, MALDI-TOF MS correctly identified 100% of *Cryptococcus* spp. in previous studies (Firacative et al., 2012; van Belkum et al., 2015; Cheng et al., 2016). Similarly, our study demonstrated that MALDI-TOF MS method is reliable, as we obtained a 100% consistency with results obtained using DNA sequencing.

The antifungal agents tested retained activity against all *Cryptococcus* isolates. Both *C. neoformans* var. *grubii* and *C. gattii* showed susceptibility to amphotericin B (MIC, 0.25–1 μg/ml). These results are consistent with previous reports (Thompson et al., 2009; Favalessa et al., 2014; Nascimento et al., 2017); however, greater MIC values are reported in the literature and were associated with treatment failure (Lozano-Chiu et al., 1998; Perkins et al., 2005).

Studies conducted previously have shown low MIC_50_ and MIC_90_ values for fluconazole against *C. neoformans* (Datta et al., 2003; Tay et al., 2006; Lia et al., 2012). In this study, the MIC_50_ and MIC_90_ values for fluconazole were 2–4 μg/ml; however, higher MIC_50_ and MIC_90_ values (4–8 and 2–128 μg/ml) have been reported for *C. neoformans* isolates (Sar et al., 2004; Favalessa et al., 2014).

High MIC values (≥64 μg/ml) have been reported for fluconazole against *C. gattii* (Sar et al., 2004; Tay et al., 2006; Soares et al., 2008; Lia et al., 2012), in contrast to this study, where we determined MIC values of ≤ 16 μg/ml. It is worth mentioning that MIC ≥16 μg/ml is believed to be the resistance cutoff for fluconazole; this value was observed from a single *C. gattii* (*C. deuterogattii*) isolate in this study. This observation brings the resistance concerns to attention and suggests that FLC might lose its potency, especially if used as a single treatment.

The new azoles (voriconazole, posaconazole, and isavuconazole) showed high antifungal activity against all *C. neoformans* and *C. gattii* strains, with low MIC values of ≤0.5 μg/ml, which is consistent with previous reports (Pfaller et al., 2003, 2009; Sabatelli et al., 2006; Illnait-Zaragozi et al., 2008; Torres-Rodríguez et al., 2008). Itraconazole, on the other hand, showed high activity against all isolates with low MIC values of ≤0.25, which is similar or lower than those of previous reports (Alves et al., 2001; Souza et al., 2010; Chowdhary et al., 2011; Herkert et al., 2018); however, 13 *C. neoformans* strains (14.13%) showed to be susceptible-dose dependent to itraconazole. Similarly, *C. neoformans* strains from Serbia showed to be susceptible-dose dependent to itraconazole (Arsic Arsenijevic et al., 2014). Nevertheless, itraconazole is rarely used as a single drug, especially in the treatment of cryptococcal meningitis, due to low concentration in the cerebrospinal fluid.

Studies conducted in several parts of the world have shown MIC values of ≤64 μg/ml for 5-FC against *C. neoformans* and *C. gattii* (Thompson et al., 2009; Chowdhary et al., 2011; Arsic Arsenijevic et al., 2014). In this study, all isolates were susceptible to 5-FC with MIC values ≤2 μg/ml; however, one *C. neoformans* strain (1.09%) was susceptible-dose dependent to 5-FC.

We observed no statistically significant difference between *C. neoformans* var. *grubii* and *C. gattii*, nor between *C. gattii s.s.* and *C. deuterogattii* isolates against all seven antifungal drugs (*p* > 0.05). Overall, according to CLSI M27-A4, most strains in this study were of wild type, except for one that might acquire resistance to FLC. A follow-up study is required to confirm if it was of non-wild type.

In summary, cryptococcosis is a complicated disease that can be easily misdiagnosed due to the lack of specificity of the clinical manifestations. In China, healthy individuals are more prone to the infection. *C. neoformans* var. *grubii* is the most predominant strain in Guangxi. MALDI-TOF MS is a rapid and reliable method to identify *Cryptococcus* spp. All isolates showed no resistance to commonly used antifungal drugs and were highly susceptible to the new triazoles. Antifungal susceptibility tests are desirable to early detect any resistant strains in order to ensure proper and successful therapy of cryptococcosis.

## Data Availability Statement

The raw data supporting the conclusions of this article will be made available by the authors, without undue reservation.

## Ethics Statement

Written informed consent was obtained from the individual(s) for the publication of any potentially identifiable images or data included in this article.

## Author Contributions

X-YL, NA-O, C-YL, and C-YH contributed to the data collection. X-YL, X-CC, B-KL, K-SP, Y-QZ, and D-YZ contributed to the laboratory work. NA-O and X-YL wrote the manuscript. W-QL and C-WC supervised and evaluated the process of the study. All authors contributed to manuscript revision and read and approved the submitted version.

## Conflict of Interest

The authors declare that the research was conducted in the absence of any commercial or financial relationships that could be construed as a potential conflict of interest.

## Publisher’s Note

All claims expressed in this article are solely those of the authors and do not necessarily represent those of their affiliated organizations, or those of the publisher, the editors and the reviewers. Any product that may be evaluated in this article, or claim that may be made by its manufacturer, is not guaranteed or endorsed by the publisher.

## Footnotes

1
http://www.ncbi.nlm.nih.gov/

## Abbreviations

FLC: fluconazole
AmB: amphotericin B
ITC: itraconazole
5-FC: 5-flucytosine
VOC: voriconazole
POS: posaconazole
ISA: isavuconazole
SDA: Sabouraud dextrose agar medium
CGB: L-canavanine -glycine-bromothymol blue
MALDI-TOF MS: matrix-assisted laser desorption ionization-time of flight mass spectrometry
ECVs: epidemiological cutoff values.
